# Modeling the Role of Peroxisome Proliferator-Activated Receptor γ and MicroRNA-146 in Mucosal Immune Responses to *Clostridium difficile*

**DOI:** 10.1371/journal.pone.0047525

**Published:** 2012-10-11

**Authors:** Monica Viladomiu, Raquel Hontecillas, Mireia Pedragosa, Adria Carbo, Stefan Hoops, Pawel Michalak, Katarzyna Michalak, Richard L. Guerrant, James K. Roche, Cirle A. Warren, Josep Bassaganya-Riera

**Affiliations:** 1 Nutritional Immunology and Molecular Medicine Laboratory, Virginia Bioinformatics Institute, Virginia Tech, Blacksburg, Virginia, United States of America; 2 Center for Modeling Immunity to Enteric Pathogens, Virginia Tech, Blacksburg, Virginia, United States of America; 3 Division of Infectious Disease and International Health, Center for Global Health, University of Virginia, Charlottesville, Virginia, United States of America; 4 Medical Informatics and Systems Division, Virginia Bioinformatics Institute, Virginia Tech, Blacksburg, Virginia, United States of America; 5 Department of Biological Sciences, Virginia Tech, Blacksburg, Virginia, United States of America; Charité, Campus Benjamin Franklin, Germany

## Abstract

*Clostridium difficile* is an anaerobic bacterium that has re-emerged as a facultative pathogen and can cause nosocomial diarrhea, colitis or even death. Peroxisome proliferator-activated receptor (PPAR) γ has been implicated in the prevention of inflammation in autoimmune and infectious diseases; however, its role in the immunoregulatory mechanisms modulating host responses to *C. difficile* and its toxins remains largely unknown. To characterize the role of PPARγ in *C. difficile*-associated disease (CDAD), immunity and gut pathology, we used a mouse model of *C. difficile* infection in wild-type and T cell-specific PPARγ null mice. The loss of PPARγ in T cells increased disease activity and colonic inflammatory lesions following *C. difficile* infection. Colonic expression of IL-17 was upregulated and IL-10 downregulated in colons of T cell-specific PPARγ null mice. Also, both the loss of PPARγ in T cells and *C. difficile* infection favored Th17 responses in spleen and colonic lamina propria of mice with CDAD. MicroRNA (miRNA)-sequencing analysis and RT-PCR validation indicated that miR-146b was significantly overexpressed and nuclear receptor co-activator 4 (NCOA4) suppressed in colons of *C. difficile*-infected mice. We next developed a computational model that predicts the upregulation of miR-146b, downregulation of the PPARγ co-activator NCOA4, and PPARγ, leading to upregulation of IL-17. Oral treatment of *C. difficile*-infected mice with the PPARγ agonist pioglitazone ameliorated colitis and suppressed pro-inflammatory gene expression. In conclusion, our data indicates that miRNA-146b and PPARγ activation may be implicated in the regulation of Th17 responses and colitis in *C. difficile*-infected mice.

## Introduction

*Clostridium difficile* typically is a harmless environmental sporulated gram-positive anaerobic bacterium [1], [2], but it has recently re-emerged as a significant enteric pathogen implicated in nosocomial diarrhea, colitis and even death, particularly after antibiotic treatment. *C. difficile* grows in the intestine of individuals with altered commensal microflora [3], [4] due to treatment with antimicrobials, immunosuppressants, cytostatic agents or proton pump inhibitors [5]. An increase in both incidence and severity of *C. difficile*-associated disease (CDAD) has been reported over the last years [6]–[8]. Previously, CDAD was a concern in older or severely ill patients, but the emergence of new hypervirulent strains such as NAP1/BI/027 has resulted in increased morbidity and mortality for other age groups in the United States, Canada and Europe [9]–[11]. The increased virulence of *C. difficile* is attributed to greater sporulation and production of binary toxins [12], [13] or to higher level of fluoroquinolone resistance [14]. Persistent or severe CDAD is currently being treated with discontinuation of the antibiotic therapy that led to the disease, and vancomycin therapy [15]. Nevertheless, these therapeutic approaches do not restore the normal microflora and are not effective in clostridial clearance, but further prolong *C. difficile* shedding and destroy beneficial gut anaerobic bacteria [15], [16]. In contrast to targeting the bacterium and its toxins directly, the better understanding of the cellular and molecular basis underlying the host response will enable the rational development of host-targeted therapeutics for CDAD.

Peroxisome proliferator-activated receptor γ (PPARγ) is a nuclear receptor and ligand-activated transcription factor involved in glucose homeostasis and lipid metabolism. PPARγ antagonizes the activity of NF-κB, STAT and AP-1. Specifically, it suppresses NF-κB [17] by stabilizing the inhibitory κB (IκB)/NF-κB [18], thereby blocking pro-inflammatory gene transcription. More importantly, activation of PPARγ modulates mucosal immune responses and is involved in the prevention of inflammatory bowel disease (IBD) in mice [17], [19], pigs [20], and humans [21], [22]. Moreover, mice with a targeted deletion of PPARγ in epithelial cells, macrophages or T cells display increased pro-inflammatory gene expression and susceptibility to colitis [23]–[25]. PPARγ also suppresses Th1 responses [19] and blocks the differentiation of CD4+ T cells into a Th17 phenotype, thus potentiating a regulatory T (Treg) cell response [26]. However, no studies are available investigating the role of PPARγ in the pathogenesis and treatment of CDAD.

Another mechanism by which colonic gene expression can be tightly regulated is microRNA (miRNA)-driven RNA interference. MiRNAs are small (∼22–24-nucleotide), non-coding, single-stranded RNA molecules that are processed from longer primary-miRNA transcripts. In the last decade, miRNAs have emerged as new potent genome regulators [27] and therapeutic targets [28]. MiRNAs are broadly found in plants, animals, viruses, and algae [29] and contribute to regulating gene expression. These molecules lead to translation inhibition of specific mRNAs depending on the type of base-pairing between the miRNA and its mRNA target [30]. In mammals, miRNA mostly affect the mRNA translation process, but mRNA target degradation also occurs. The role of miRNA has been explored in IBD and other immune-mediated diseases as a promising avenue for the discovery of novel mechanisms of pathogenesis, diagnostics, and therapeutics. Distinct miRNA expression profiles have been found in Crohn's disease and ulcerative colitis [31]. There is also mounting evidence that miRNAs contribute to orchestrate immune regulation and host responses to pathogen infections. For example, miR-146a and miR-155 are involved in the regulation of T- and B-cell development [32], their differentiation and function [33]. Mice lacking miR-155 fail to control *Helicobacter pylori* infection as a result of impaired Th1 and Th17 responses [34]. Therefore, understanding the role of miRNAs in antibacterial immune and inflammatory responses holds promise of new molecular diagnostic markers as well as novel gene therapy strategies for treating hypervirulent bacterial infections and associated immunopathologies.

This study investigates the mechanisms underlying PPARγ modulation of mucosal immune responses to *C. difficile*, including a possible relationship between nuclear receptors and miRNAs. Specifically, we applied mathematical and computational modeling approaches in combination with mouse challenge studies to study the mechanisms underlying the interactions between PPARγ activity and miRNA-146b to regulate colitis during *C. difficile* infection. Next, we investigated how either T cell-specific deletion or pharmacological activation of PPARγ modulate colonic inflammatory cytokines and effector Th17 responses to *C. difficile* infection in mice. Our data indicate that T cell PPARγ prevents colitis and down-modulates effector T cell responses in mice with CDAD and suggest a potential crosstalk between miRNAs and the PPAR γ pathway.

## Materials and Methods

### Ethics Statement

All experimental procedures were approved by the Institutional Animal Care and Use Committee (IACUC) of Virginia Tech and met or exceeded requirements of the Public Health Service/National Institutes of Health and the Animal Welfare Act. The IACUC approval ID for the study was 10-087-VBI.

### Animal procedures

C57BL/6J wild type mice were bred in our laboratory animal facilities. Tissue-specific PPARγ null mice were generated as described previously [17], [35], [36]. The tail and colonic genotypes of mice were determined by PCR analysis as described previously [17], [37]. Specifically, we used CD4-Cre+ mice lacking PPARγ in T cells [38] and MMTV-Cre+ mice with a deletion in epithelial and hematopietic cells [17]. Mice were maintained at the experimental facilities at Virginia Tech.

### Antibiotic pretreatment prior to the bacterial challenge

Previous studies have demonstrated that three days of pre-treatment with a mixture of antibiotics in the drinking water is sufficient to disrupt the intestinal microflora and allows *C. difficile* infection [39]. The antibiotic mixture consisted of colistin 850 U/mL (which corresponds to 4.2 mg/kg), gentamicin 0.035 mg/mL (which corresponds to 3.5 mg/kg), metronidazole 0.215 mg/mL (which corresponds to 21.5 mg/kg) and vancomycin 0.045 mg/mL (which corresponds to 4.5 mg/kg). The mixture was prepared and added to the drinking water for a 3-day pre-treatment period. A control group that received no antibiotics was also included. Following the treatment all mice were given regular autoclaved water for 2 days and all mice including the control group received a single dose of clindamycin (32 mg/kg) intraperitoneally 1 day before *C. difficile* challenge.

### Clostridium difficile mouse challenge studies

On day 5 of the study mice were challenged intragastrically with *Clostridium difficile* strain VPI 10463 (ATCC 43255). To optimize the infection dose for further studies, we conducted a dose-response experiment using the following *C. difficile* infectious doses: 10^5^, 10^6^ and 10^7^ cfu/mouse. The highest dose was used for the following challenges. In some experiments mice received pioglitazone at 70 mg/kg by oral gavage once daily starting 3 days before the infection date and until the necropsy day. Mice were weighed and scored daily to assess mortality and the presence of diarrhea and other general disease symptoms (e.g., piloerection, hunchback position).

### Histopathology

Segments of colon (3 cm of the anatomic middle of the colon) were fixed in 10% buffered neutral formalin. Samples were embedded in paraffin, sectioned (6 μm), and then stained with hematoxylin and eosin for histological examination. Tissue slides were examined with an Olympus microscope (Olympus America). Images were captured using the FlashBus FBG software (Integral Technologies) and processed in Adobe Photoshop Elements 2.0 (Adobe Systems). The different tissue segments were graded with a compounded histological score, including the extent of: 1) crypt damage and regeneration; 2) metaplasia/hyperplasia; 3) lamina propria vascular changes; 4) submucosal changes; and 5) presence of inflammatory infiltrates. The sections were graded with a range from 0 to 4 for each of the previous categories and data were analyzed as a normalized compounded score.

### Analyses of miRNA-seq

Next-generation sequencing allows the sequencing of miRNA molecules and simultaneous quantification of their expression levels. We used Illumina deep sequencing to survey miRNA profiles of colons from uninfected and *C. difficile*-infected mice with most distinct phenotypes. All reads were mapped against the mouse reference genome. To determine putative gene targets of miRNA, the EMBL-EBI Microcosm v5 database was used. Additionally, precursor and mature sequences were retrieved from MirBase v16 [40] and entered into microRNAminer [41]. Following characterization of miRNA expression profiles with Partek Genomics Suite, pair-wise analyses between infected and uninfected colonic samples were conducted to identify the most differentially expressed miRNA. Data was submitted to NCBI's GEO database (Accession Number GSE39235).

### Functional correlation between the expression of miR-146b and some of its potential targets

Potential targets for miR-146b and the regulatory pathways that are expected to be regulated were identified in the literature [42]. The list of candidates mRNA targets was retrieved from the MicroCosm Targets database Version 5 (http://www.ebi.ac.uk/enright-srv/microcosm), formely known as mirBase::Targets [40], that uses the miRanda algorithm [43] to identify potential binding sites for a given miRNA in gene sequences. Among these findings, we focused on mRNAs we expected to be involved in the pathogenesis of *C. difficile* by regulating genes at acute stages of the disease. Knowing that miRNA can induce a significant degradation of its target and assuming that evolution progressively selected inverse regulation of expression of mRNAs and their specific miRNAs, we selected nuclear receptor coactivator 4 (NCOA4), a miR-146b target for differential expression testing using qRT-PCR between mice uninfected or mice infected with 107 cfu of *C. difficile*. NCOA4 was selected for further analyses since it is a well-known activator of PPARγ.

### Quantitative real-time RT-PCR

Total RNA was isolated from mouse colons using a Qiagen RNA Isolation Mini kit according to the manufacturer's instructions. Total RNA (1 μg) was used to generate a cDNA template using an iScript cDNA Synthesis kit (Bio-Rad). The total reaction volume was 20 μL, with the reaction incubated as follows in an MJ MiniCycler: 5 min at 25°C, 30 min at 52°C, 5 min at 85°C, and hold at 4°C. PCR was performed on the cDNA using Taq DNA polymerase (Invitrogen) under previously described conditions [44]. Each gene amplicon was purified with the MiniElute PCR Purification kit (Qiagen) and quantified both on an agarose gel by using a DNA mass ladder (Promega) and with a nanodrop. These purified amplicons were used to optimize real-time PCR conditions and to generate standard curves in the real-time PCR assay. Primers were designed using Oligo 6 software. Primer concentrations and annealing temperatures were optimized for the iCycler iQ system (Bio-Rad) for each set of primers using the system's gradient protocol. PCR efficiencies were maintained between 92 and 105% and correlation coefficients >0.98 for each primer set during optimization and also during the real-time PCR of sample DNA. cDNA concentrations for genes of interest were examined by real-time qPCR using an iCycler IQ System and the iQ SYBR green supermix (Bio-Rad). A standard curve was generated for each gene using 10-fold dilutions of purified amplicons starting at 5 pg of cDNA and used later to calculate the starting amount of target cDNA in the unknown samples. SYBR green I is a general double-stranded DNA intercalating dye and may therefore detect nonspecific products and primer/dimers in addition to the amplicon of interest. To determine the number of products synthesized during the real-time PCR, a melting curve analysis was performed on each product. Real-time PCR was used to measure the starting amount of nucleic acid of each unknown sample of cDNA on the same 96-well plate.

Mmu-miR-146b* expression was analyzed with quantitative RT-PCR using TaqMan MicroRNA Assays from Applied Biosystems. Two small nucleolar RNAs, snoRNA202 and snoRNA234, were used as endogenous normalizers for target miR-146b*. A total of 100 ng/sample of RNA was used for cDNA synthesis using the ABI TaqMan MicroRNA Reverse Transcription Kit and the manufacturer protocol. To test for genomic DNA and reaction contamination, two types of negative controls were used for PCR: reverse transcriptase-omitted products and blanks (DEPC-treated water). No amplification was observed in any of the negative controls. TaqMan Universal Master Mix II, the ABI StepOnePlus RT-PCR System and the instrument default cycling conditions were used for PCR. There were 6–8 biological replicates per group, with each RNA sample assayed twice in separate RT reactions. The threshold cycle (C_T_) ratios between the target miRNA and the average endogenous control were calculated, arcsin-transformed, and one-way repeated measures ANOVA in R (v. 2.14.0) was used to test differences between treatments and PCR replicates.

### Immunophenotyping

Colon samples were processed for lamina propria lymphocyte (LPL) isolation. Specifically, cells (6×10^5^ cells/well) were seeded into 96 well-plates, centrifuged at 4°C at 2000 rpm for 3 minutes, and washed with PBS containing 5% serum and 0,09% sodium azide (FACS buffer). Cells were then incubated for T cell assessment with fluorochrome-conjugated primary antibodies to T cell markers. Cells were first incubated with AF700-labeled anti mouse CD45, PECy5-labeled anti mouse CD3 and PECy7-labeled anti mouse CD4. Cells were then fixed and permeabilized with Cytofix-Cytoperm solution (Pharmingen) and incubated with PerCpCy5.5-labeled anti mouse IL-17 and PE-labeled anti mouse RORγt. Cells were resuspended in 0.2 mL of FACS buffer. Data acquisition was computed with a BD LSR II flow cytometer and analysis performed with FACS Diva software (BD Pharmingen).

### In silico simulations of the involvement of miRNA-146b and PPARγ in modulating colonic host responses to C. difficile infection

**Figure 1:**
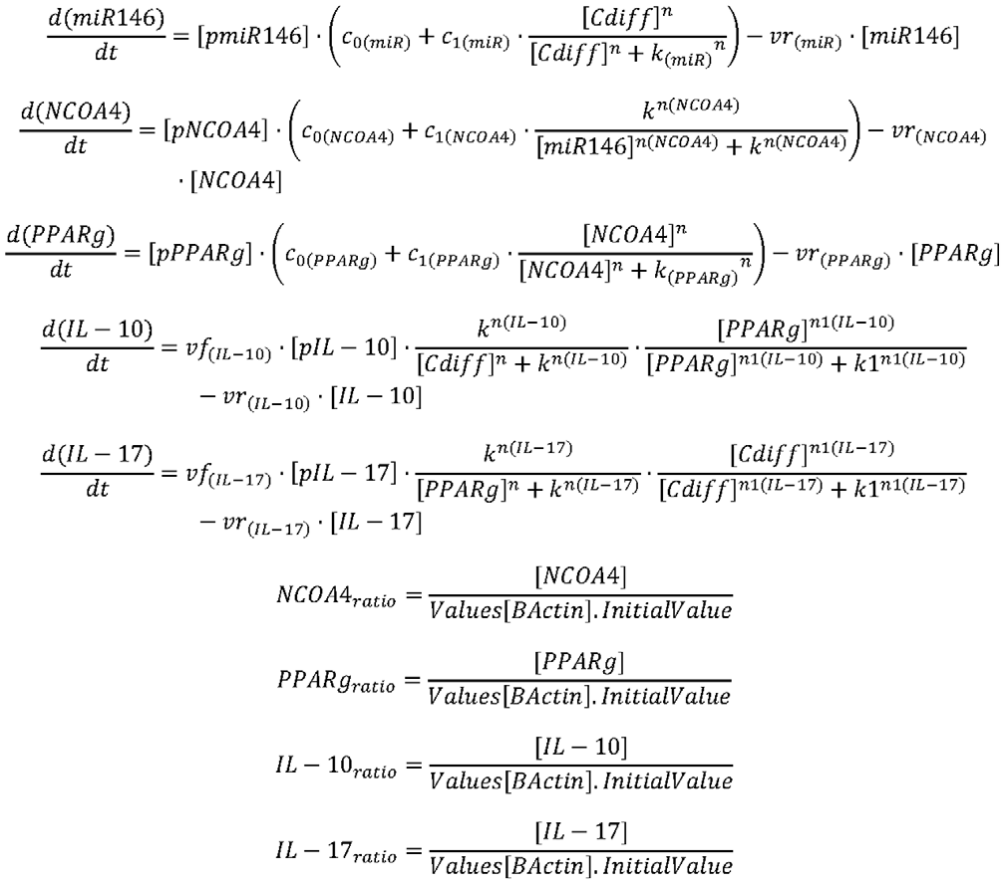
Equations controlling dynamics of the *Clostridium difficile* infection model. Ordinary Differential Equations (ODE) triggering activation and inhibition of the different reactions in the model. Briefly, mass action and Hill functions were used to reproduce reaction behaviors *in silico* based on initial molecule concentrations.

Based on the results obtained in the mouse challenge studies, an ordinary differential equation-based computational model was developed describing the molecular dynamics of some key cytokines and transcription factors involved in *C. difficile* infection. Although modeling approaches cannot replace traditional experimentation, the construction of such computational model synthesized, organized and integrated all the concepts and mechanisms studied, facilitating a more systematic hypothesis-generation process. Overall, our modeling process involved: 1) creation of a structural network using Cell Designer; 2) parameter estimation based on published or newly generated experimental data using Complex Pathway Simulator (COPASI); and 3) *in silico* experimentation. We constructed a network model with five dynamic variables representing miR-146b, NCOA4, PPARγ, interleukin 17 (IL-17) and IL-10, plus an external input: the infectious dose of *C. difficile*. The network model was constructed by using CellDesigner [45], a software package that enables users to describe molecular interactions using a well-defined and consistent graphical notation. Modeling was performed using COPASI (http://www.modelingimmunity.org/) [46]. Both COPASI and CellDesigner are Systems Biology Markup Language (SBML) compliant, thus, machine and human readable. The CellDesigner-generated network was imported into COPASI where rate laws were adjusted to create the ordinary differential equations (Figure 1). The results of the parameter estimation using Particle Swarm showed a good fitting between the experimental data and predicted values computationally estimated by COPASI (Table 1). These values were then implemented in the reactions and rate laws to adjust the dynamics of the model. COPASI was then used to run sensitivity analysis and *in silico* time-course studies.

**Table 1:** Model fitting performed by using COPASI's global parameter estimation.

Fitted Value	Objective Value	Root Mean Square	Error Mean	Error Mean Std. Deviation
miR-146b	1.41E-23	2.65E-12	6.73E-13	2.56E-12
NCOA4_ratio	1.19E-19	2.44E-10	−2.42E-10	2.96E-11
PPARγ_ratio	2.95E-20	1.21E-10	−1.17E-10	3.37E-11
IL10_ratio	7.05E-11	5.94E-06	−6.36E-08	5.94E-06
IL17_ratio	0.431748	0.464623	0.328534	0.328542

A species is fitted computationally using experimental data and simulation algorithms. The objective value is the value that the modeling software targets based on the experimental data and the computational simulation.

### Statistical analyses

We performed Analysis of Variance (ANOVA) to determine the significance of the model by using the general linear model procedure of SAS (SAS Institute) as previously described [17]. Specifically, we examined the main effect of genotype, treatment, and their interaction when necessary. Differences of *P*<0.05 were considered significant. Data were expressed as the means ± SE of the mean.

## Results

### Increasing doses of C. difficile infection correlate with increasing CDAD and colonic inflammatory lesions

We first performed a dose-response study to identify the optimal infectious dose that results in greater inflammatory responses in wild type mice and found that clinical signs of disease appeared as early as 24 hours post-infection following challenge with *C. difficile* strain VPI10463. Increasing infectious doses of *C. difficile* from 10^5^ to 10^7^ cfu corresponded with greater disease severity and colonic inflammatory lesions. Colon, cecum, spleen and MLN were scored for inflammation-related gross pathology lesions during the necropsy. All *C. difficile*-infected mice presented gross pathological lesions in all the examined tissues when compared to uninfected mice (Figure S1). Colonic histopathological analyses showed increased epithelial erosion, leukocytic infiltration and mucosal thickness, with more severe inflammatory lesions corresponding to increasing infectious doses of *C. difficile*. In addition, microscopic examination revealed extensive areas of necrosis of the mucosa and submucosal edema (Figure S2).

### C. difficile upregulates colonic pro-inflammatory cytokine expression

**Figure 2:**
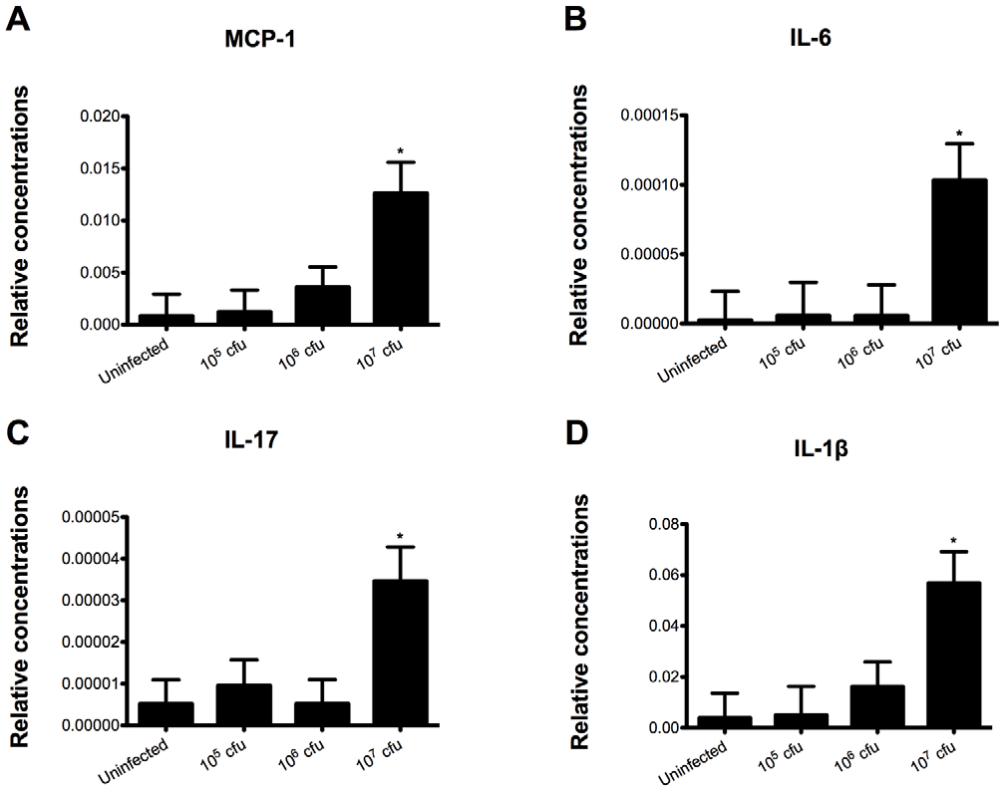
*Clostridium difficile* infection modulates colonic gene expression in mice. Colonic expression of monocyte chemotactic Protein 1 (MCP-1) (A), interleukin 6 (IL-6) (B), interleukin 17 (IL-17) (C) and interleukin 1β (IL-1β) (D were assessed by real-time quantitative RT-PCR in mice infected with *C. difficile* (n = 10). Data are represented as mean ± standard error. Points with an asterisk are significantly different when compared to the control group (*P*<0.05).

RNA was extracted from colon and real time RT-PCR was performed to examine the effect of *C. difficile* infection on colonic gene expression. Mice challenged with 10^7^ cfu of *C. difficile* had a significant increase in monocyte chemoattractant protein 1 (MCP-1), IL-6, IL-17 and IL-1β when compared to all the other groups, indicating that the bacterial challenge induced a strong pro-inflammatory response in the gut (Figure 2). Although mice infected with 10^5^ and 10^6^ cfu had a higher disease activity score than the control mice, no increase in the colonic expression of such cytokines was seen in these groups, possibly due to a counterbalance mediated by a parallel regulatory response at lower doses of infection.

### Infected mice overexpress miR-146b

**Figure 3:**
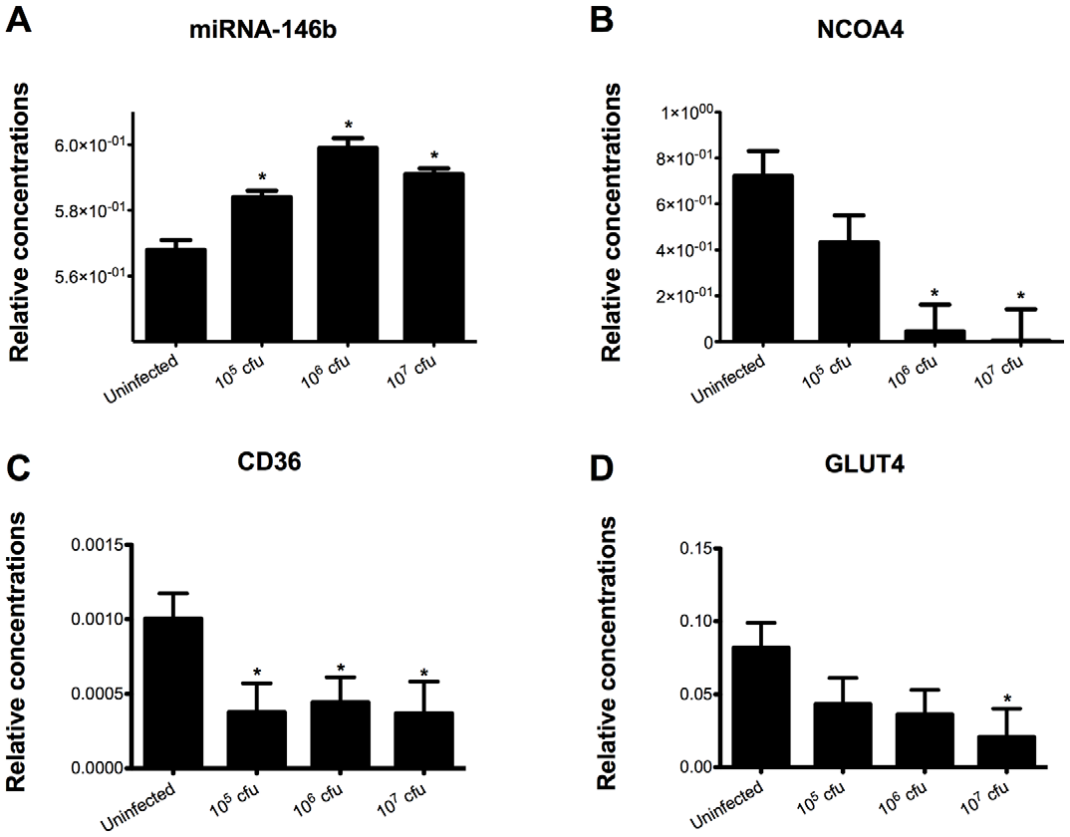
Effect of *Clostridium difficile* infection on the colonic expression of miR-146b and target genes NCOA4, CD36 and GLUT4 mRNA in mice. Colonic expression of miRNA-146b (A) as well as NCOA4 (B), CD36 (C) and GLUT4 (D) were assessed by real-time quantitative RT-PCR in mice infected with *C. difficile* (n = 10). Data are represented as mean ± standard error. Points with an asterisk are significantly different when compared to the control group (*P*<0.05).

MiRNA profiles of *C. difficile*-infected and uninfected mice were determined by Illumina sequencing. Specifically, a total of 454 miRNA types were detected among 8,902,783 Illumina reads from the six samples (3 non-infected and 3 infected with 107 cfu) multiplexed within two lanes. Three miRNAs were significantly overexpressed within infection: miR-146b, miR-1940, and miR-1298 (FDR *P*<0.05) (Figure S3). The sequencing results were validated by real-time RT-PCR. Our data showed an upregulation of miR-146b correlating with the dose of *C. difficile* infection. NCOA4 was computationally predicted as a target of miRNA-146b based on thermodynamics. In order to begin to validate such prediction, co-expression of miRNA-146b and NCOA4 within the colon and differential expression of NCOA4 with increasing doses of infection was assessed by RT-PCR. Interestingly, a significant decrease in the expression of colonic NCOA4 was found with increasing *C. difficile* infectious doses. NCOA4 is a coactivator molecule that interacts with the PPAR γ complex and facilitates its activation. Therefore, the decrease in NCOA4 expression results in a reduction of PPAR γ activation. In line with the suppression of NCOA4 expression we found that PPAR γ target genes CD36 and GLUT4 were significantly downregulated in colons of *C. difficile*-infected mice (Figure 3), indicating that colonic PPARγ activity is suppressed in mice infected with *C. difficile*.

### Computational modeling of host responses to C. difficile infection

**Figure 4:**
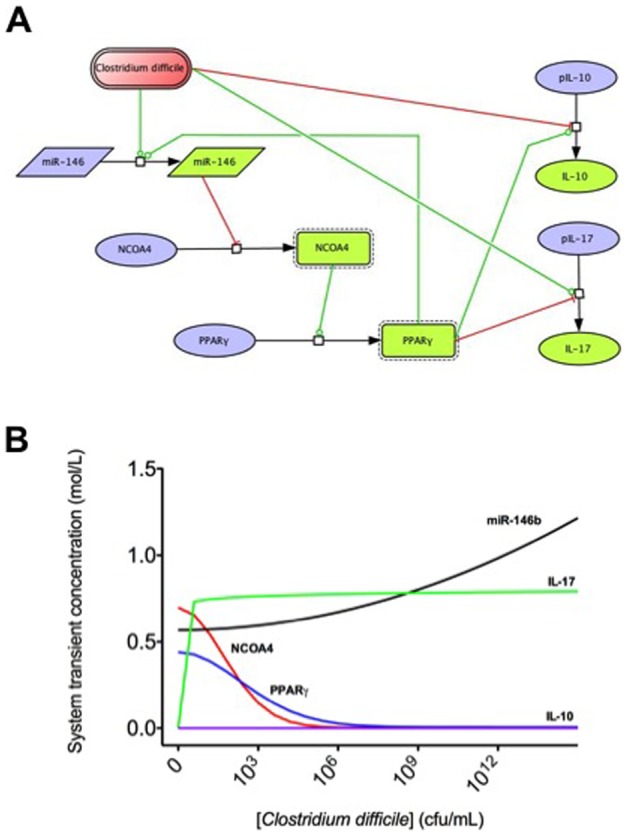
Computational modeling of mucosal immune responses to *Clostridium difficile* infection. CellDesigner-based illustration of the Complex Pathway SImulator model of the model for Clostridium difficile immune response (A). The model represents the interaction between *C. difficile*, miRNA-146, nuclear receptor coactivator 4 (NCOA4), peroxisome proliferator-activated receptor γ (PPAR γ), interleukin 10 (IL-10) and interleukin 17 (IL-17) in Systems Biology Markup Language format. Inhibition is represented in red and activation in green. COPASI steady state scan showing the variation on the species concentrations with increasing computational concentration of *C. difficile* (B). In silico simulations show how increasing concentrations of *C. difficile* increase miRNA-146b levels, thus decreasing NCOA4 and PPAR γ. In line with the experimental data, IL-17 expression also increases with the infection.

To further integrate and characterize the potential interactions occurring between *C. difficile*, miR-146b and PPARγ, we developed a computational and mathematical model of the colonic gene expression changes occurring in the colon following *C difficile* infection. This network was constructed based on our experimental findings and literature information (Figure 4A). By using this model, we explored *in silico* the mechanisms by which *C. difficile* modulates the expression of effector and inflammatory cytokines. Our computational simulation predicts an upregulation of miR-146b, and IL-17 and a down-regulation of NCOA4 and PPARγ in colons of mice after infection with *C. difficile* (Figure 4B).

### The loss of PPARγ in T cells significantly increased CDAD and colitis following C. difficile infection

**Figure 5:**
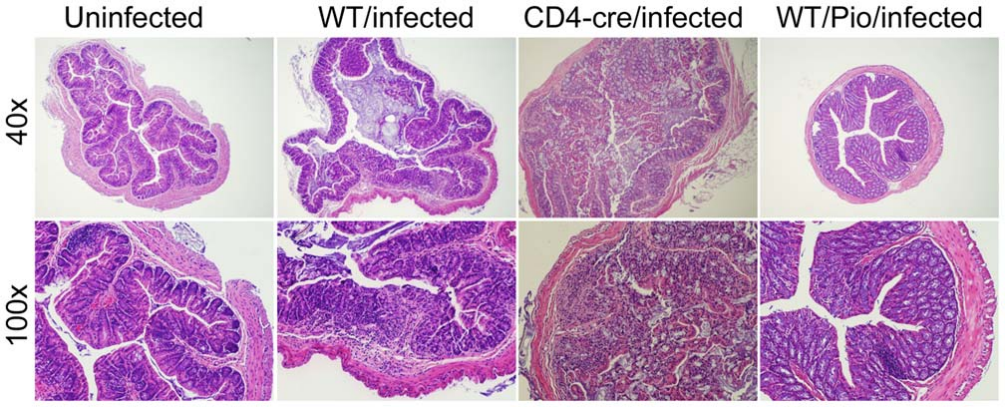
Impact of the loss of PPARγ in T cells and pharmacological activation of PPARγ colonic inflammatory lesions in *Clostridium difficile*-infected mice. Representative photomicrographs of colons of uninfected (A and E), infected wild type mice (B and F), infected CD4cre+ mice (C and G) and infected wild-type mice treated orally with pioglitazone (70 mg/kg) (D and H) (n = 8). Original magnification at 40× (top panel) and 100× (bottom panel).

Since our data indicates that the PPARγ pathway is downregulated at the colonic mucosa during *C. difficile* infection, we assessed the impact of the loss of PPARγ in epithelial and hematopoietic cells on *C. difficile* infection-associated weight loss. Our results show a more dramatic weight loss and more accentuated inflammatory lesions in tissue-specific PPARγ null mice following infection with *C. difficile* (data not shown). In a follow-up study we determined the impact of T cell-specific PPARγ deletion in the inflammatory response and disease caused by this facultative anaerobic bacterium. T cell-specific PPARγ null (i.e., CD4cre+) mice had higher disease activity scores than wild type mice did. They also showed a 20% body weight loss by day 4 post-infection which resulted in 30% of mortality (Figure S4). Moreover, infected CD4cre+ mice had more severe colonic inflammatory lesions (Figure 5), suggesting that PPARγ expression in T cells plays and important role in ameliorating CDAD in mice. Uninfected CD4cre+ mice did not show any difference when compared to the uninfected WT group (data not shown). On the other hand, mice treated with the PPARγ agonist pioglitazone (70 mg/kg) had reduced inflammatory lesions, colonic histopathology (Figure 4), and had lower levels of colonic inflammatory mediators when compared to untreated mice infected with *C. difficile* (Figure S5). Also, uninfected WT mice treated with pioglitazone did not differ from uninfected and untreated group.

### The loss of T cell PPARγ causes upregulation of colonic MCP-1 and IL-17 and downregulation of IL-10 in C. difficile-infected mice

**Figure 6:**
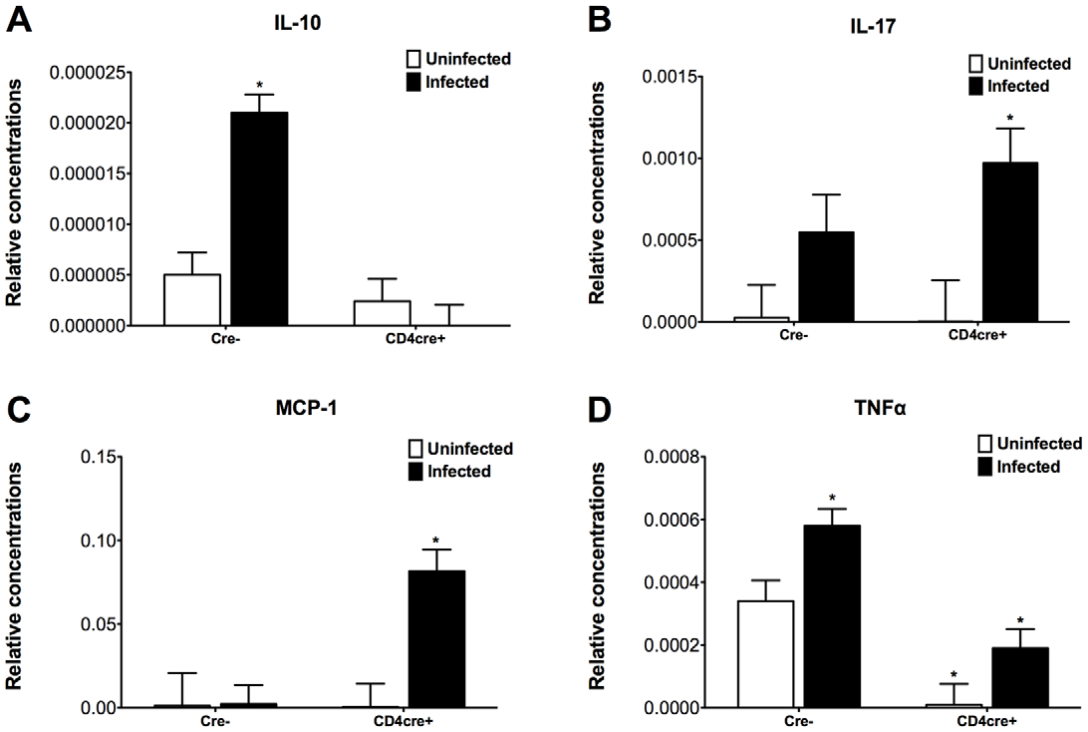
The loss of PPARγ in T cells regulates colonic cytokine expression of mice infected with *Clostridium difficile*. Colonic expression of interleukin 10 (IL-10) (A), interleukin 17 (IL-17) (B), monocyte chemoattractant protein 1 (MCP-1) (C) and tumor necrosis factor (TNF-α) (D) were assessed by real-time quantitative RT-PCR in wild type and T cell PPARγ null mice infected with *C. difficile* (n = 8). Data are represented as mean ± standard error. Points with an asterisk are significantly different when compared to the wild type control group (*P*<0.05).

**Figure 7:**
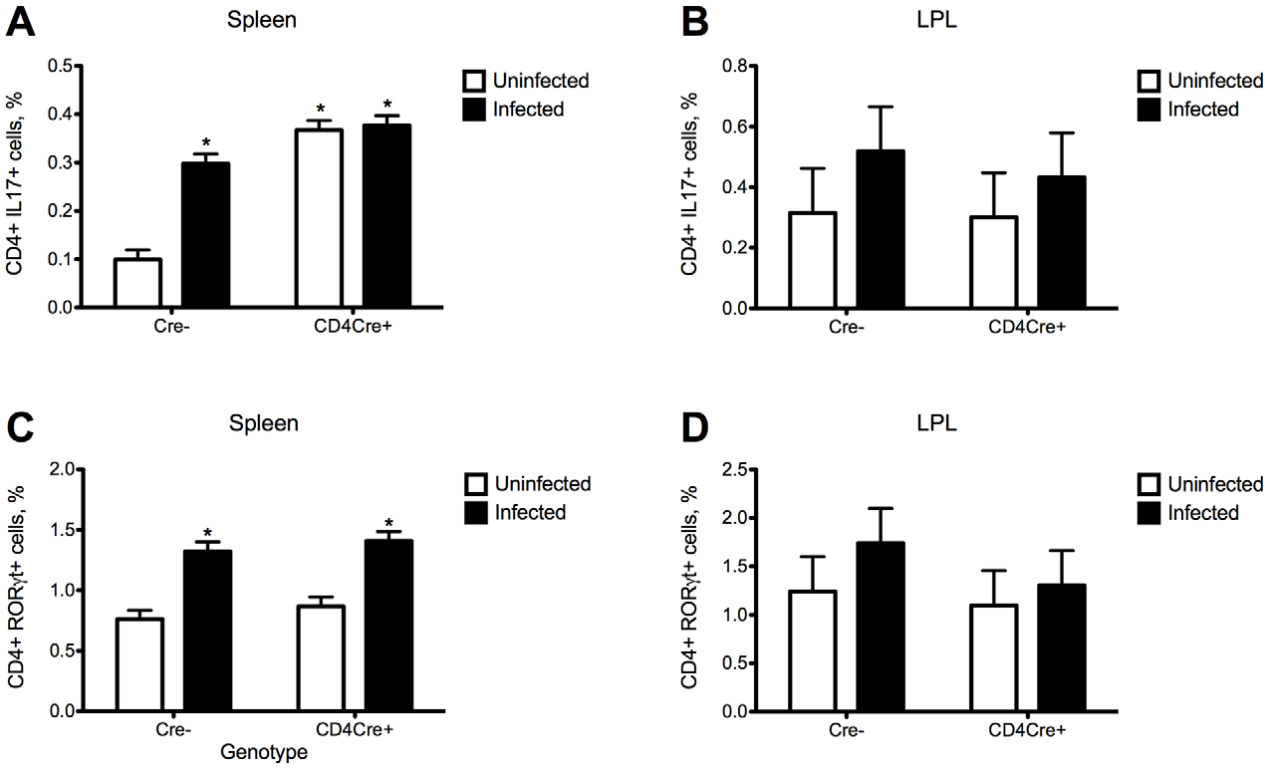
The loss of PPARγ in T cells and *Clostridium difficile* infection enhances Th17 responses in spleen and lamina propria of mice. Splenocytes and lamina propria lymphocytes from wild type and T cell PPARγnull mice infected with *C. difficile* (n = 6) were immunophenotyped to identify immune cell subsets by flow cytometry. Data are represented as mean ± standard error. Points with an asterisk are significantly different when compared to the control group (*P*<0.05).

Colonic IL-10 expression was upregulated in colonic wild type mice when compared to those of T cell-specific PPARγ null (CD4cre+) mice, indicating that the deficiency of PPARγ in T cells abrogated the ability of *C. difficile* infection to induce IL-10 expression. In addition, the deficiency of PPARγ in T cells caused an upregulation of colonic MCP-1, IL-17 and tumor necrosis factor α (TNF-α) mRNA expression (Figure 6). Flow cytometric analyses of immune cell populations indicates an increased percentages of IL-17+ and RORγt+ CD4+ T cells following the infection as well as increased percentages of Th17 cells in spleens of T cell-specific PPARγ null mice (Figure 7).

## Discussion

*Clostridium difficile* is the most common cause of nosocomial infectious diarrhea in the U.S. An increase in the severity and incidence of *C. difficile* infections has been reported in the last few years [47]. Moreover, the increased report of cases in healthy people without traditional risk factors is alarming since it suggests a greater pathogenicity of circulating strains [48], [49]. Hence, elucidating the cellular and molecular basis controlling the host-*C. difficile* interactions is important to discover new broad-based, host-targets therapeutic approaches to control the disease. This report presents fully integrated *in vivo* and computational approaches for studying the mechanisms involved in the regulation of host responses to *C. difficile*. We constructed a network model with five dynamical variables representing miR-146b, NCOA4, PPARγ, IL-17 and IL-10, plus an external input: the infectious dose of C. difficile. The computational simulations predicted an overexpression of miR-146b following C. difficle infection, resulting in decreased concentrations of NCOA4, which in turn failed to activate PPARγ. In addition, colonic gene expression analyses demonstrated an upregulation of IL-6 and IL-17 in *C. difficile*-infected mice, suggesting either enhanced Th17 responses or IL-17 production by other mucosal cell types such as paneth cells or γδ T cells. Th17 responses are implicated in immune-mediated diseases and immune responses to some extracellular pathogens. Flow cytometric analyses of immune cell populations indicated increased percentages of IL-17+ and RORγt+ CD4+ T cells in spleens of infected mice, suggesting that *C. difficile* infection enhanced Th17 responses.

PPARγ plays a crucial role during CD4+ T cell differentiation by down-modulating effector and enhancing regulatory responses [19], [38], and thereby contributing to the maintenance of tissue homeostasis. Furthermore, PPARγ agonists have proven therapeutic and prophylactic efficacy in IBD [17], [19], [20]. To determine whether the loss of the PPARγ gene in T cells worsened CDAD, we challenged wild-type and T cell-specific PPARγ null mice with *C. difficile*. The bacterial infection induced a colonic mucosal IL-10-driven anti-inflammatory response as well as an effector response characterized by the production of IL-17 and MCP-1 in wild-type mice. However, the loss of PPARγ in T cells abrogated the ability of *C. difficile* to induce IL-10 expression in the colon, without affecting the overproduction of IL-17, which has been implicated in immune-mediated diseases such as IBD. Flow cytometry analysis confirmed that both *C. difficile* infection and the loss of PPARγ favor Th17 responses. The therapeutic value of PPARγ activation in CDAD was further demonstrated by the significant amelioration of CDAD and suppression of colitis in mice treated with pioglitazone, a full PPAR γ agonist.

Pharmacological manipulation of miRNA has been postulated as a novel and viable therapeutic approach to modulate the host inflammatory response to minimize pathology without negatively affecting pathogen clearance or the ability to mount a protective immune response. Mature miRNAs are incorporated into the RNA-induced silencing complex where they typically recognize and bind to sequences in the 3′ untranslated regions, leading to suppression of translation and/or degradation of mRNA. There is accumulating evidence that miRNA play a critical role in immunity and inflammation [31]–[33]. For instance, distinct miRNA expression profiles have been found in Crohn's disease and ulcerative colitis [31]. By comparing miRNA profiles from *C. difficile*-infected and uninfected mice, we found that miR-146b, miR-1940, and miR-1298 were overexpressed in colons of *C. difficile*-infected mice. We focused our efforts on the miRNA-146 family (miRNA-146a/b) in this study since we validated its upregulation in the colon of C. difficile-infected mice by using RT-PCR. In addition, miRNA-146 is expressed in leukocytes and its function is clearly linked to innate immunity and inflammation [50], [51]. miRNA-146 regulatory circuit improves TLR4 and cytokine signaling in response to microbial components and proinflammatory cytokines [52], [53]. Moreover, it is involved in the regulation of T- and B-cell development [32], [33], differentiation and function [33]. To further understand the role of miRNA-146b during *C. difficile* infection, a list of mRNA potential targets for such miRNA was retrieved from miRBase [40]. Notably, one of the co-activators facilitating the transcriptional activities of the ligand-activated PPARγ, NCOA4, was a predicted target of miRNA-146b. Indeed, gene expression analyses using qRT-PCR demonstrated co-expression of miRNA-146b and NCOA4 in colons of *C. difficile*-infected mice and a negative correlation between expression of miR-146b and its target NCOA4 along with increasing doses of *C. difficile*, suggesting a potential inhibition of NCOA4 by miR-146b resulting in suppressed PPARγ activity, as measured by suppressed expression of PPAR γ-responsive genes (i.e., CD36 and Glut4) in *C. difficile*-infected mice. Thus, miRNAs become promising therapeutic targets once the functional consequences of miRNAs alteration are completely elucidated. Also, future studies should examine more direct therapeutic approaches to prevent overexpression of miRNA-146 during CDAD.

In conclusion, we have used loss-of-function approaches in combination with pharmacological activation of PPARγ and computational modeling to investigate the critical role of PPARγ in regulating immune responses and disease severity following *C. difficile* infection. Our data suggests that overexpression of miRNA-146b in the colon might exacerbate inflammatory responses by suppressing PPARγ activity through a mechanism possibly involving suppression of NCOA4, a co-activator molecule required for activation of PPARγ.

## Supporting Information

Figure S1 **Effect of infection with** ***Clostridium difficile*** **strain VPI 10463 on macroscopic inflammation-related lesions in C57BL/6J wild-type mice.** Colon (A), cecum (B), spleen (C) and mesenteric lymph nodes (MLN) (D) were macroscopically scored for inflammation during the necropsy (n = 10). Data are represented as mean ± standard error. Points with an asterisk are significantly different when compared to the control group (*P*<0.05).
(TIFF)

Figure S2 **Effect of infection with** ***Clostridium difficile*** **strain VPI 10463 on microscopic lesions observed following a 4-day challenge.** Representative photomicrographs of colons of uninfected (A and D), infected with 10^6^ colony-forming units (cfu) of *C. difficile* (B and E) and infected with 10^7^ cfu of *C. difficile* (C and F) (n = 10). Original magnification at 40× (top panel) and 100× (bottom panel).
(TIF)

Figure S3 **Effect of infection with** ***Clostridium difficile*** **strain VPI 10463 on miRNA differential expression in C57BL/6J wild-type mice.** miRNA-seq heatmap illustrating the clustering results of *C. difficile*-infected (T) and uninfected control (C) mice (n = 3).
(TIF)

FigureS4 **Effect of the genotype and treatment on the body weight loss, disease activity index and histologic lesions in the colons of mice infected with** ***Clostridium difficile*** **strain VPI 10463.** Mice were weighed (A) and scored (B) daily for mortality and morbidity and the presence of diarrhea and other symptoms (n = 8). All colonic specimens underwent blinded histological examination and were scored 0–4 on mucosal wall thickening (C&E) and leukocyte infiltration (D&F). Data are represented as mean ± standard error. Points with an asterisk are significantly different when compared to the control group (*P*<0.05).
(TIF)

Figure S5 **Effect of the oral pioglitazone administration on the colon gene expression of mice infected with** ***Clostridium difficile*** **strain VPI 10463.** Colonic expression of interleukin 1β (IL-1β) (A), monocyte chemoattractant protein 1 (MCP-1) (B) and interleukin 17 (IL-17) (C) were assessed by real-time quantitative RT-PCR in *C. difficile* infected wild type mice treated with pioglitazone (n = 8). Data are represented as mean ± standard error. Points with an asterisk are significantly different when compared to the control group (*P*<0.05).
(TIFF)

## References

[1] Vaishnavi C (2010) Clinical spectrum & pathogenesis of Clostridium difficile associated diseases. Indian J Med Res 131: 487–499. 20424299

[2] Hookman P, Barkin JS (2009) Clostridium difficile associated infection, diarrhea and colitis. World J Gastroenterol 15: 1554–1580. 19340897 10.3748/wjg.15.1554 PMC2669939

[3] Bartlett JG, Onderdonk AB, Cisneros RL, Kasper DL (1977) Clindamycin-associated colitis due to a toxin-producing species of Clostridium in hamsters. J Infect Dis 136: 701–705. 915343 10.1093/infdis/136.5.701

[4] George RH, Symonds JM, Dimock F, Brown JD, Arabi Y, et al (1978) Identification of Clostridium difficile as a cause of pseudomembranous colitis. Br Med J 1: 695. 630301 10.1136/bmj.1.6114.695 PMC1603073

[5] Kim JW, Lee KL, Jeong JB, Kim BG, Shin S, et al (2010) Proton pump inhibitors as a risk factor for recurrence of Clostridium-difficile-associated diarrhea. World J Gastroenterol 16: 3573–3577. 20653067 10.3748/wjg.v16.i28.3573 PMC2909558

[6] Lim PL, Barkham TM, Ling LM, Dimatatac F, Alfred T, et al (2008) Increasing incidence of Clostridium difficile-associated disease, Singapore. Emerg Infect Dis 14: 1487–1489. 18760029 10.3201/eid1409.070043 PMC2603103

[7] Khanna S, Pardi DS (2010) The growing incidence and severity of Clostridium difficile infection in inpatient and outpatient settings. Expert Rev Gastroenterol Hepatol 4: 409–416. 20678014 10.1586/egh.10.48

[8] Zilberberg MD, Shorr AF, Kollef MH (2008) Increase in adult Clostridium difficile-related hospitalizations and case-fatality rate, United States, 2000–2005. Emerg Infect Dis 14: 929–931. 18507904 10.3201/eid1406.071447 PMC2600276

[9] Khanna S, Pardi DS, Aronson SL, Kammer PP, Orenstein R, et al (2012) The epidemiology of community-acquired Clostridium difficile infection: a population-based study. Am J Gastroenterol 107: 89–95. 22108454 10.1038/ajg.2011.398 PMC3273904

[10] Kuehn BM (2011) Scientists seek strategies to prevent Clostridium difficile infections. JAMA 306: 1849–1850. 22045758 10.1001/jama.2011.1569

[11] Barbut F, Jones G, Eckert C (2011) Epidemiology and control of Clostridium difficile infections in healthcare settings: an update. Curr Opin Infect Dis 24: 370–376. 21505332 10.1097/QCO.0b013e32834748e5

[12] Akerlund T, Persson I, Unemo M, Noren T, Svenungsson B, et al (2008) Increased sporulation rate of epidemic Clostridium difficile Type 027/NAP1. J Clin Microbiol 46: 1530–1533. 18287318 10.1128/JCM.01964-07 PMC2292905

[13] Merrigan M, Venugopal A, Mallozzi M, Roxas B, Viswanathan VK, et al (2010) Human hypervirulent Clostridium difficile strains exhibit increased sporulation as well as robust toxin production. J Bacteriol 192: 4904–4911. 20675495 10.1128/JB.00445-10 PMC2944552

[14] Pawlowski SW, Archbald-Pannone L, Carman RJ, Alcantara-Warren C, Lyerly D, et al (2009) Elevated levels of intestinal inflammation in Clostridium difficile infection associated with fluoroquinolone-resistant C. difficile. J Hosp Infect 73: 185–187. 19709778 10.1016/j.jhin.2009.05.013 PMC2743747

[15] McFarland LV (2005) Alternative treatments for Clostridium difficile disease: what really works? J Med Microbiol 54: 101–111. 15673502 10.1099/jmm.0.45753-0

[16] Ryan A, Lynch M, Smith SM, Amu S, Nel HJ, et al (2011) A role for TLR4 in Clostridium difficile infection and the recognition of surface layer proteins. PLoS Pathog 7: e1002076. 21738466 10.1371/journal.ppat.1002076 PMC3128122

[17] Bassaganya-Riera J, Reynolds K, Martino-Catt S, Cui Y, Hennighausen L, et al (2004) Activation of PPAR gamma and delta by conjugated linoleic acid mediates protection from experimental inflammatory bowel disease. Gastroenterology 127: 777–791. 15362034 10.1053/j.gastro.2004.06.049

[18] Li M, Pascual G, Glass CK (2000) Peroxisome proliferator-activated receptor gamma-dependent repression of the inducible nitric oxide synthase gene. Mol Cell Biol 20: 4699–4707. 10848596 10.1128/mcb.20.13.4699-4707.2000 PMC85890

[19] Hontecillas R, Bassaganya-Riera J (2007) Peroxisome proliferator-activated receptor gamma is required for regulatory CD4+ T cell-mediated protection against colitis. J Immunol 178: 2940–2949. 17312139 10.4049/jimmunol.178.5.2940

[20] Bassaganya-Riera J, Hontecillas R (2006) CLA and n-3 PUFA differentially modulate clinical activity and colonic PPAR-responsive gene expression in a pig model of experimental IBD. Clin Nutr 25: 454–465. 16698153 10.1016/j.clnu.2005.12.008

[21] Lewis JD, Lichtenstein GR, Deren JJ, Sands BE, Hanauer SB, et al (2008) Rosiglitazone for active ulcerative colitis: a randomized placebo-controlled trial. Gastroenterology 134: 688–695. 18325386 10.1053/j.gastro.2007.12.012 PMC2276587

[22] Bassaganya-Riera J, Hontecillas R, Horne WT, Sandridge M, Herfarth HH, et al. (2012) Conjugated linoleic acid modulates immune responses in patients with mild to moderately active Crohn's disease. Clin Nutr. 10.1016/j.clnu.2012.03.002 22521469

[23] Guri AJ, Mohapatra SK, Horne WT, 2nd, Hontecillas R, Bassaganya-Riera J (2010) The role of T cell PPAR gamma in mice with experimental inflammatory bowel disease. BMC Gastroenterol 10: 60. 20537136 10.1186/1471-230X-10-60 PMC2891618

[24] Hontecillas R, Horne WT, Climent M, Guri AJ, Evans C, et al (2011) Immunoregulatory mechanisms of macrophage PPAR-gamma in mice with experimental inflammatory bowel disease. Mucosal Immunol 4: 304–313. 21068720 10.1038/mi.2010.75 PMC3049196

[25] Mohapatra SK, Guri AJ, Climent M, Vives C, Carbo A, et al (2010) Immunoregulatory actions of epithelial cell PPAR gamma at the colonic mucosa of mice with experimental inflammatory bowel disease. PLoS One 5: e10215. 20422041 10.1371/journal.pone.0010215 PMC2857885

[26] Klotz L, Burgdorf S, Dani I, Saijo K, Flossdorf J, et al (2009) The nuclear receptor PPAR gamma selectively inhibits Th17 differentiation in a T cell-intrinsic fashion and suppresses CNS autoimmunity. J Exp Med 206: 2079–2089. 19737866 10.1084/jem.20082771 PMC2757877

[27] Lewis BP, Burge CB, Bartel DP (2005) Conserved seed pairing, often flanked by adenosines, indicates that thousands of human genes are microRNA targets. Cell 120: 15–20. 15652477 10.1016/j.cell.2004.12.035

[28] Hassan T, McKiernan PJ, McElvaney NG, Cryan SA, Greene CM (2012) Therapeutic modulation of miRNA for the treatment of proinflammatory lung diseases. Expert Rev Anti Infect Ther 10: 359–368. 22397568 10.1586/eri.11.175

[29] Papaioannou MD, Nef S (2010) microRNAs in the testis: building up male fertility. J Androl 31: 26–33. 19875496 10.2164/jandrol.109.008128

[30] Bartel DP (2004) MicroRNAs: genomics, biogenesis, mechanism, and function. Cell 116: 281–297. 14744438 10.1016/s0092-8674(04)00045-5

[31] Dalal SR, Kwon JH (2010) The Role of MicroRNA in Inflammatory Bowel Disease. Gastroenterol Hepatol (N Y) 6: 714–722. 21437020 PMC3033542

[32] Chen CZ, Li L, Lodish HF, Bartel DP (2004) MicroRNAs modulate hematopoietic lineage differentiation. Science 303: 83–86. 14657504 10.1126/science.1091903

[33] Sonkoly E, Stahle M, Pivarcsi A (2008) MicroRNAs and immunity: novel players in the regulation of normal immune function and inflammation. Semin Cancer Biol 18: 131–140. 18291670 10.1016/j.semcancer.2008.01.005

[34] Oertli M, Engler DB, Kohler E, Koch M, Meyer TF, et al (2011) MicroRNA-155 is essential for the T cell-mediated control of Helicobacter pylori infection and for the induction of chronic Gastritis and Colitis. J Immunol 187: 3578–3586. 21880981 10.4049/jimmunol.1101772

[35] Akiyama TE, Sakai S, Lambert G, Nicol CJ, Matsusue K, et al (2002) Conditional disruption of the peroxisome proliferator-activated receptor gamma gene in mice results in lowered expression of ABCA1, ABCG1, and apoE in macrophages and reduced cholesterol efflux. Mol Cell Biol 22: 2607–2619. 11909955 10.1128/MCB.22.8.2607-2619.2002 PMC133709

[36] Wagner KU, McAllister K, Ward T, Davis B, Wiseman R, et al (2001) Spatial and temporal expression of the Cre gene under the control of the MMTV-LTR in different lines of transgenic mice. Transgenic Res 10: 545–553. 11817542 10.1023/a:1013063514007

[37] Cui Y, Miyoshi K, Claudio E, Siebenlist UK, Gonzalez FJ, et al (2002) Loss of the peroxisome proliferation-activated receptor gamma (PPARgamma ) does not affect mammary development and propensity for tumor formation but leads to reduced fertility. J Biol Chem 277: 17830–17835. 11884400 10.1074/jbc.M200186200

[38] Wohlfert EA, Nichols FC, Nevius E, Clark RB (2007) Peroxisome proliferator-activated receptor gamma (PPARgamma) and immunoregulation: enhancement of regulatory T cells through PPARgamma-dependent and -independent mechanisms. J Immunol 178: 4129–4135. 17371968 10.4049/jimmunol.178.7.4129

[39] Chen X, Katchar K, Goldsmith JD, Nanthakumar N, Cheknis A, et al (2008) A mouse model of Clostridium difficile-associated disease. Gastroenterology 135: 1984–1992. 18848941 10.1053/j.gastro.2008.09.002

[40] Griffiths-Jones S, Saini HK, van Dongen S, Enright AJ (2008) miRBase: tools for microRNA genomics. Nucleic Acids Res 36: D154–158. 17991681 10.1093/nar/gkm952 PMC2238936

[41] Artzi S, Kiezun A, Shomron N (2008) miRNAminer: a tool for homologous microRNA gene search. BMC Bioinformatics 9: 39. 18215311 10.1186/1471-2105-9-39 PMC2258288

[42] Garbacki N, Di Valentin E, Huynh-Thu VA, Geurts P, Irrthum A, et al (2011) MicroRNAs profiling in murine models of acute and chronic asthma: a relationship with mRNAs targets. PLoS One 6: e16509. 21305051 10.1371/journal.pone.0016509 PMC3030602

[43] John B, Enright AJ, Aravin A, Tuschl T, Sander C, et al (2004) Human MicroRNA targets. PLoS Biol 2: e363. 15502875 10.1371/journal.pbio.0020363 PMC521178

[44] Hontecillas R, Wannemeulher MJ, Zimmerman DR, Hutto DL, Wilson JH, et al (2002) Nutritional regulation of porcine bacterial-induced colitis by conjugated linoleic acid. J Nutr 132: 2019–2027. 12097686 10.1093/jn/132.7.2019

[45] Funahashi A, Jouraku A, Matsuoka Y, Kitano H (2007) Integration of CellDesigner and SABIO-RK. In Silico Biol 7: S81–90. 17822394

[46] Hoops S, Sahle S, Gauges R, Lee C, Pahle J, et al (2006) COPASI–a COmplex PAthway SImulator. Bioinformatics 22: 3067–3074. 17032683 10.1093/bioinformatics/btl485

[47] Kuntz JL, Chrischilles EA, Pendergast JF, Herwaldt LA, Polgreen PM (2011) Incidence of and risk factors for community-associated Clostridium difficile infection: a nested case-control study. BMC Infect Dis 11: 194. 21762504 10.1186/1471-2334-11-194 PMC3154181

[48] Blondeau JM (2009) What have we learned about antimicrobial use and the risks for Clostridium difficile-associated diarrhoea? J Antimicrob Chemother 63: 238–242. 19028718 10.1093/jac/dkn477

[49] Kyne L, Sougioultzis S, McFarland LV, Kelly CP (2002) Underlying disease severity as a major risk factor for nosocomial Clostridium difficile diarrhea. Infect Control Hosp Epidemiol 23: 653–659. 12452292 10.1086/501989

[50] Moschos SA, Williams AE, Perry MM, Birrell MA, Belvisi MG, et al (2007) Expression profiling in vivo demonstrates rapid changes in lung microRNA levels following lipopolysaccharide-induced inflammation but not in the anti-inflammatory action of glucocorticoids. BMC Genomics 8: 240. 17640343 10.1186/1471-2164-8-240 PMC1940008

[51] Schmidt WM, Spiel AO, Jilma B, Wolzt M, Muller M (2009) In vivo profile of the human leukocyte microRNA response to endotoxemia. Biochem Biophys Res Commun 380: 437–441. 19284987 10.1016/j.bbrc.2008.12.190

[52] Lederhuber H, Baer K, Altiok I, Sadeghi K, Herkner KR, et al (2011) MicroRNA-146: tiny player in neonatal innate immunity? Neonatology 99: 51–56. 20616571 10.1159/000301938

[53] Taganov KD, Boldin MP, Chang KJ, Baltimore D (2006) NF-kappaB-dependent induction of microRNA miR-146, an inhibitor targeted to signaling proteins of innate immune responses. Proc Natl Acad Sci U S A 103: 12481–12486. 16885212 10.1073/pnas.0605298103 PMC1567904

