# Supplementary figures and images for: Modeling the Role of Peroxisome Proliferator-Activated Receptor γ and MicroRNA-146 in Mucosal Immune Responses to Clostridium difficile
Source: PLoS One. 2012 Oct 11;7(10):e47525. doi: 10.1371/journal.pone.0047525 (PMC3469550; doi:10.1371/journal.pone.0047525)

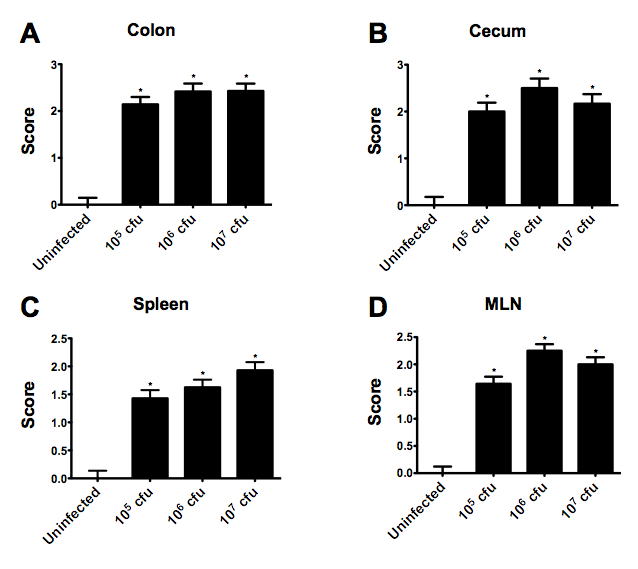

Supplement: Figure S1 — Effect of infection with Clostridium difficile strain VPI 10463 on macroscopic inflammation-related lesions in C57BL/6J wild-type mice. Colon (A), cecum (B), spleen (C) and mesenteric lymph nodes (MLN) (D) were macroscopically scored for inflammation during the necropsy (n = 10). Data are represented as mean ± standard error. Points with an asterisk are significantly different when compared to the control group (P<0.05). (TIFF) [file pone.0047525.s001.tiff]

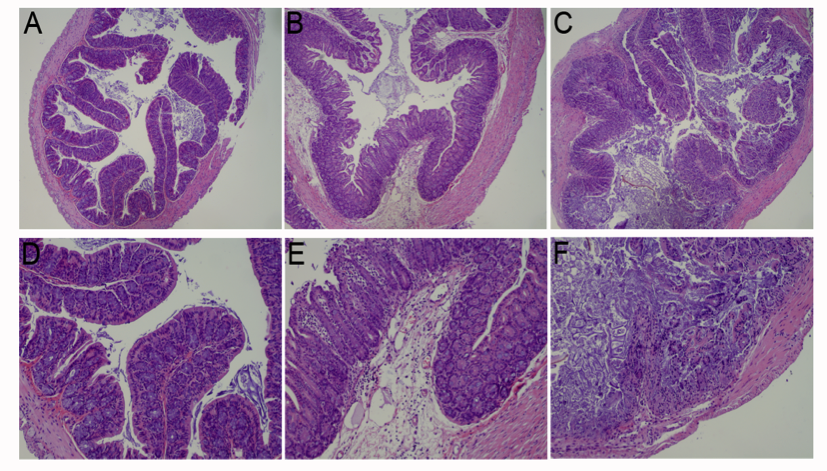

Supplement: Figure S2 — Effect of infection with Clostridium difficile strain VPI 10463 on microscopic lesions observed following a 4-day challenge. Representative photomicrographs of colons of uninfected (A and D), infected with 106 colony-forming units (cfu) of C. difficile (B and E) and infected with 107 cfu of C. difficile (C and F) (n = 10). Original magnification at 40× (top panel) and 100× (bottom panel). (TIF) [file pone.0047525.s002.tif]

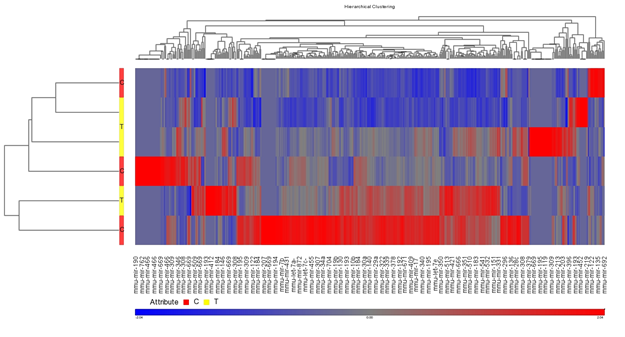

Supplement: Figure S3 — Effect of infection with Clostridium difficile strain VPI 10463 on miRNA differential expression in C57BL/6J wild-type mice. miRNA-seq heatmap illustrating the clustering results of C. difficile-infected (T) and uninfected control (C) mice (n = 3). (TIF) [file pone.0047525.s003.tif]

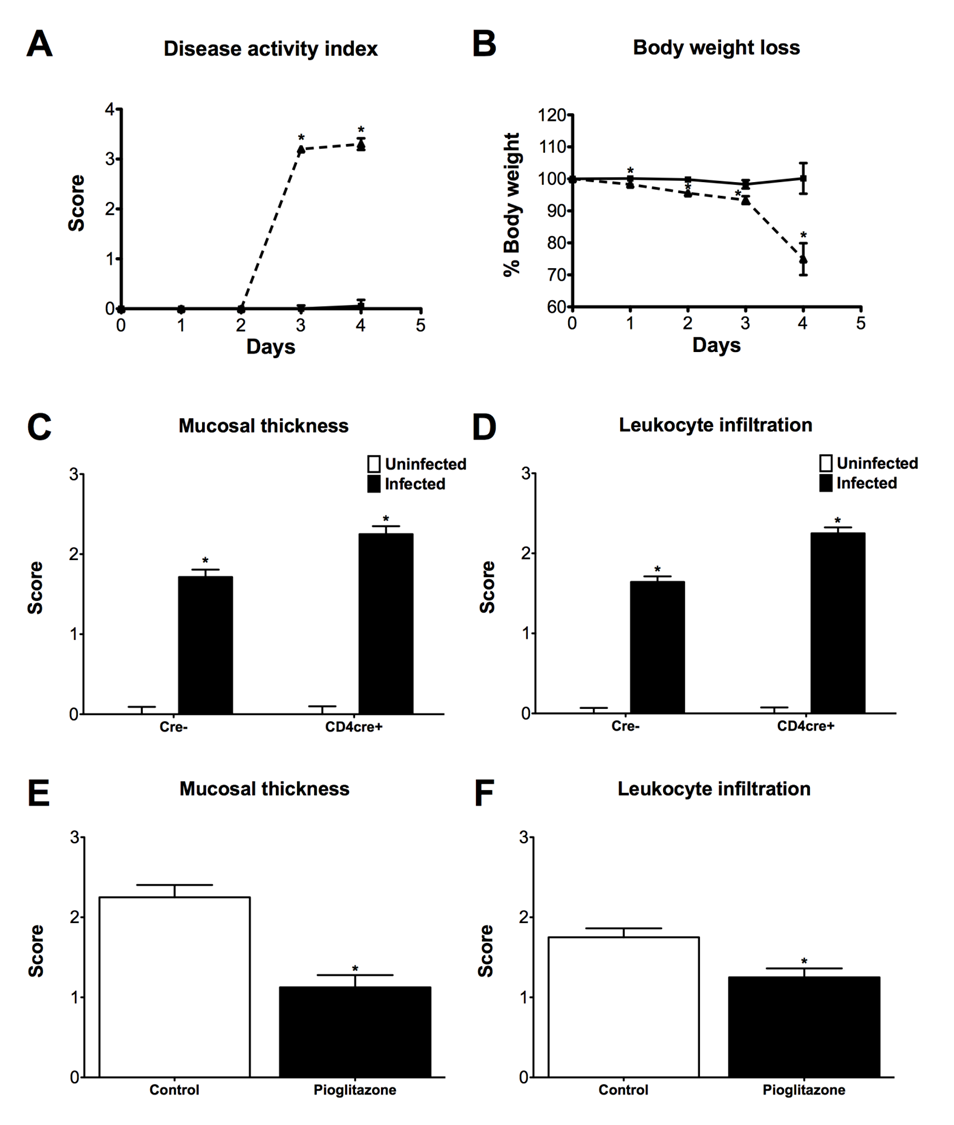

Supplement: FigureS4 — Effect of the genotype and treatment on the body weight loss, disease activity index and histologic lesions in the colons of mice infected with Clostridium difficile strain VPI 10463. Mice were weighed (A) and scored (B) daily for mortality and morbidity and the presence of diarrhea and other symptoms (n = 8). All colonic specimens underwent blinded histological examination and were scored 0–4 on mucosal wall thickening (C&E) and leukocyte infiltration (D&F). Data are represented as mean ± standard error. Points with an asterisk are significantly different when compared to the control group (P<0.05). (TIF) [file pone.0047525.s004.tif]

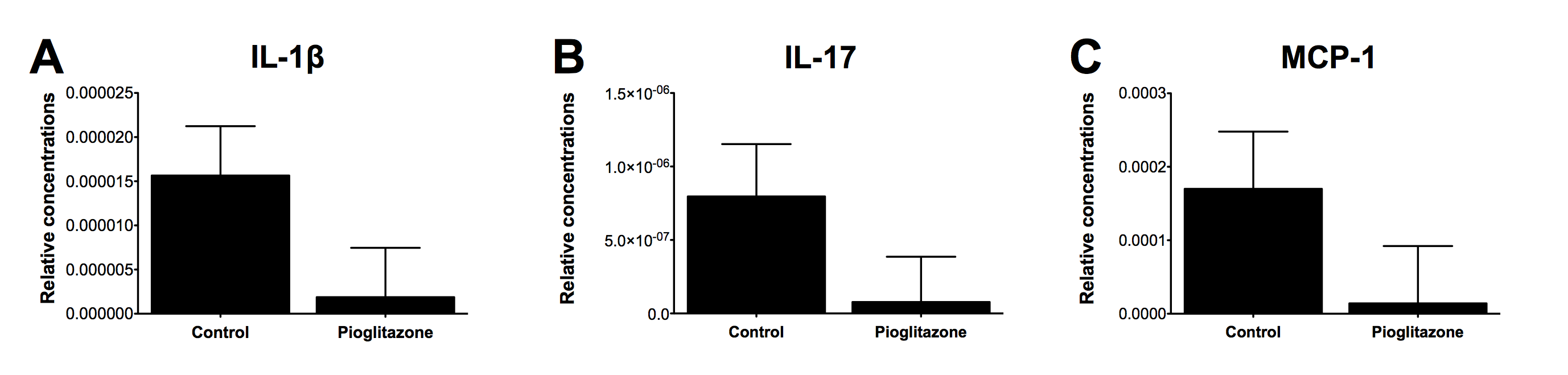

Supplement: Figure S5 — Effect of the oral pioglitazone administration on the colon gene expression of mice infected with Clostridium difficile strain VPI 10463. Colonic expression of interleukin 1β (IL-1β) (A), monocyte chemoattractant protein 1 (MCP-1) (B) and interleukin 17 (IL-17) (C) were assessed by real-time quantitative RT-PCR in C. difficile infected wild type mice treated with pioglitazone (n = 8). Data are represented as mean ± standard error. Points with an asterisk are significantly different when compared to the control group (P<0.05). (TIFF) [file pone.0047525.s005.tiff]
